# 3,5-Dimethyl­pyrazolium 3,5-dinitro­salicylate

**DOI:** 10.1107/S1600536812041906

**Published:** 2012-10-13

**Authors:** Shuaishuai Wei, Shouwen Jin, Zhaofeng Hu, Yong Zhou, Yingping Zhou

**Affiliations:** a Tianmu College of ZheJiang A & F University, Lin’An 311300, People’s Republic of China

## Abstract

In the title mol­ecular salt, C_5_H_9_N_2_^+^·C_7_H_3_N_2_O_7_^−^, the roughly planar anion (r.m.s. deviation = 0.120 Å) has been deprotonated at the phenol group. An intra­molecular O—H⋯O hydrogen bond in the anion generates an *S*(6) ring. In the crystal, the components are linked by cation-to-anion N—H⋯O and N—H⋯(O,O) hydrogen bonds, generating [010] double chains. Weak C—H⋯O inter­actions consolidate the packing.

## Related literature

For a related structure and background to hydrogen-bonding inter­actions, see: Jin *et al.* (2010 ▶). For another related structure, see: Smith *et al.* (2011 ▶).
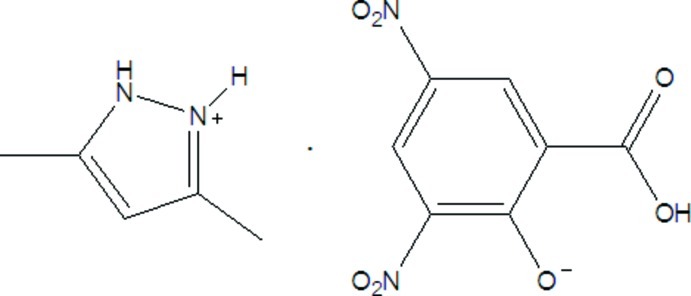


## Experimental

### 

#### Crystal data

C_5_H_9_N_2_^+^·C_7_H_3_N_2_O_7_^−^
*M* *_r_* = 324.26
Monoclinic, 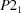

*a* = 8.1183 (7) Å
*b* = 6.0636 (5) Å
*c* = 14.1453 (11) Å
β = 91.904 (1)°
*V* = 695.93 (10) Å^3^
*Z* = 2
Mo *K*α radiation
μ = 0.13 mm^−1^
*T* = 293 K
0.40 × 0.27 × 0.11 mm

#### Data collection

Bruker SMART CCD diffractometer
Absorption correction: multi-scan (*SADABS*; Bruker, 2002 ▶) *T*_min_ = 0.959, *T*_max_ = 0.986
3523 measured reflections
2301 independent reflections
1659 reflections with *I* > 2σ(*I*)
*R*_int_ = 0.040

#### Refinement

*R*[*F* ^2^ > 2σ(*F* ^2^)] = 0.047
*wR*(*F* ^2^) = 0.105
*S* = 1.02
2301 reflections
208 parameters
1 restraint
H-atom parameters constrained
Δρ_max_ = 0.16 e Å^−3^
Δρ_min_ = −0.18 e Å^−3^

### 

Data collection: *SMART* (Bruker, 2002 ▶); cell refinement: *SAINT* (Bruker, 2002 ▶); data reduction: *SAINT*; program(s) used to solve structure: *SHELXS97* (Sheldrick, 2008 ▶); program(s) used to refine structure: *SHELXL97* (Sheldrick, 2008 ▶); molecular graphics: *SHELXTL* (Sheldrick, 2008 ▶); software used to prepare material for publication: *SHELXTL*.

## Supplementary Material

Crystal structure: contains datablock(s) global, I. DOI: 10.1107/S1600536812041906/hb6958sup1.cif

Structure factors: contains datablock(s) I. DOI: 10.1107/S1600536812041906/hb6958Isup2.hkl

Supplementary material file. DOI: 10.1107/S1600536812041906/hb6958Isup3.cml

Additional supplementary materials:  crystallographic information; 3D view; checkCIF report

## Figures and Tables

**Table 1:** Hydrogen-bond geometry (Å, °)

*D*—H⋯*A*	*D*—H	H⋯*A*	*D*⋯*A*	*D*—H⋯*A*
N1—H1⋯O7^i^	0.86	2.09	2.859 (4)	148
N1—H1⋯O1^i^	0.86	2.16	2.809 (4)	132
N2—H2⋯O3	0.86	1.88	2.684 (3)	156
O2—H2*A*⋯O1	0.82	1.72	2.481 (3)	154
C1—H1*A*⋯O7^i^	0.96	2.32	3.166 (5)	147
C5—H5*B*⋯O4^ii^	0.96	2.49	3.414 (5)	160
C10—H10⋯O6^iii^	0.93	2.48	3.379 (4)	164

Symmetry codes: (i) 
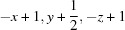
; (ii) 
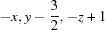
; (iii) 
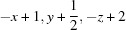
.

**Fig. 1.**
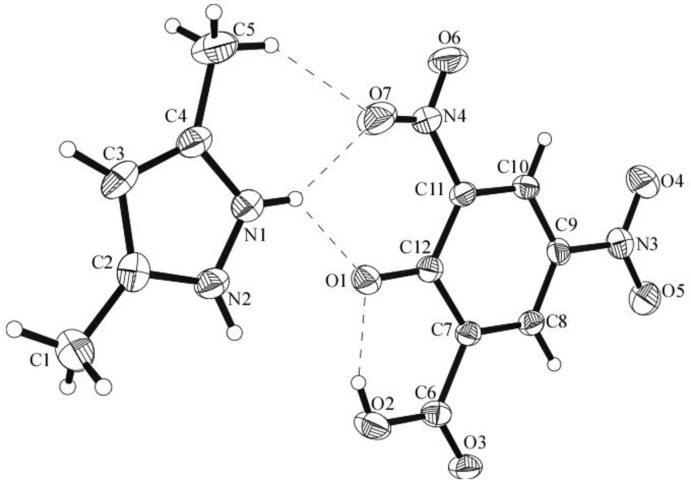
The structure of the title compound, showing displacement ellipsoids drawn at the 30% probability level.

**Fig. 2.**
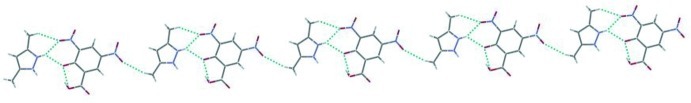
The one-dimensional chain formed through the CH3—O interaction.

## supplementary crystallographic information

### Comment

Organic acid-base adducts based on hydrogen bonding are a research field receiving great attention in recent years. As an extension of our study concentrating on hydrogen bonded assembly of organic acid and organic base (Jin *et al.*, 2010), herein we report the crystal structure of 3,5-dimethylpyrazolium 3,5-dinitrosalicylate.

The crystal of the title compound of the formula C_12_H_12_N_4_O_7_ was obtained by recrystallization of the mixture of 3,5-dimethylpyrazole and 3,5-dinitrosalicylic acid acid from the MeOH solution.

In this case (Fig. 1) it is the phenol H that has been deprotonated. The C—O distance 1.284 (4) Å concerning the phenolate is similar to the proton transfer compound bearing the 3,5-dinitrosalicylate in which only the phenol group has been deprotonated (Smith *et al.*, 2011). The C—O distances O(2)—C(6), 1.300 (4) Å, O(3)—C(6), 1.223 (4) Å; in the COOH show characteristic C—O, and C=O distances which are also confirming the reliability of adding H atoms experimentally by different electron density onto O atoms.

One anion is bonded to one cation *via* N—H···O, and CH_3_—O associations to form a heteroadduct.

For the presence of these interactions, there are close joint motifs with descriptors of *R*_2_1(6), and *R*_1_2(6). The usual intramolecular hydrogen bond is found between the phenolate and the carboxyl group to exhibit a S_1_1(6) graph. The heteroadducts were linked together by the CH_3_—O association between the methyl group of the pyrazole and the 5-nitro group with C—O distance of 3.415 Å to form one-dimensional chain running along the direction that made an angle of *ca* 60° with the *a* axis (Fig. 2). The chains were further stacked along the direction that is perpendicular with its extending direction *via* the interchain N-π interaction between the 5-nitro group and the phenyl ring of the anion with N—*Cg* distance of 3.236 Å to form two-dimensional sheet extending parallel to the *ab* plane. The sheets were further stacked along the *c* axis direction by the CH—O, N—H···O, and O—H···O associations to form three-dimensional ABAB layer network structure. Herein the chains at adjacent layers intersect at an angle of *ca* 120° with each other.

### Experimental

A solution of 3,5-dimethyl pyrazole (19.2 mg, 0.2 mmol) in 5 ml of MeOH was added to a MeOH solution (6 ml) containing 3,5-dinitrosalicylic acid (22.8 mg, 0.1 mmol) under continuous stirring. The solution was stirred for about 1 h at room temperature, then the solution was filtered into a test tube. The solution was left standing at room temperature for several days, yellow blocks were isolated after slow evaporation of the solution in air at ambient temperature. The crystals were collected and dried in air to give the title compound.

### Refinement

The absolute structure could not be determined in the present refinement. Hydrogen atoms attached to the C atoms were placed in calculated positions with d(C—H) = 0.93–0.96 Å. Positions of the hydrogen atoms at the NH, and COOH groups were located from the Fourier difference syntheses and refined independently. All *U*_iso_ values were restrained on *U*_eq_ values of the parent atoms.

### Crystal data

**Table:**

C_5_H_9_N_2_^+^·C_7_H_3_N_2_O_7_^−^	*F*(000) = 336
*M**_r_* = 324.26	*D*_x_ = 1.547 Mg m^−^^3^
Monoclinic, *P*2_1_	Mo *K*α radiation, λ = 0.71073 Å
*a* = 8.1183 (7) Å	Cell parameters from 1025 reflections
*b* = 6.0636 (5) Å	θ = 2.5–22.6°
*c* = 14.1453 (11) Å	µ = 0.13 mm^−^^1^
β = 91.904 (1)°	*T* = 293 K
*V* = 695.93 (10) Å^3^	Block, colorless
*Z* = 2	0.40 × 0.27 × 0.11 mm

### Data collection

**Table:**

Bruker SMART CCD diffractometer	2301 independent reflections
Radiation source: fine-focus sealed tube	1659 reflections with *I* > 2σ(*I*)
Graphite monochromator	*R*_int_ = 0.040
ω scans	θ_max_ = 25.0°, θ_min_ = 2.5°
Absorption correction: multi-scan (*SADABS*; Bruker, 2002)	*h* = −9→9
*T*_min_ = 0.959, *T*_max_ = 0.986	*k* = −7→7
3523 measured reflections	*l* = −16→12

### Refinement

**Table:**

Refinement on *F*^2^	Primary atom site location: structure-invariant direct methods
Least-squares matrix: full	Secondary atom site location: difference Fourier map
*R*[*F*^2^ > 2σ(*F*^2^)] = 0.047	Hydrogen site location: inferred from neighbouring sites
*wR*(*F*^2^) = 0.105	H-atom parameters constrained
*S* = 1.02	*w* = 1/[σ^2^(*F*_o_^2^) + (0.0405*P*)^2^] where *P* = (*F*_o_^2^ + 2*F*_c_^2^)/3
2301 reflections	(Δ/σ)_max_ < 0.001
208 parameters	Δρ_max_ = 0.16 e Å^−^^3^
1 restraint	Δρ_min_ = −0.18 e Å^−^^3^

### Special details

**Table:**

Geometry. All e.s.d.'s (except the e.s.d. in the dihedral angle between two l.s. planes) are estimated using the full covariance matrix. The cell e.s.d.'s are taken into account individually in the estimation of e.s.d.'s in distances, angles and torsion angles; correlations between e.s.d.'s in cell parameters are only used when they are defined by crystal symmetry. An approximate (isotropic) treatment of cell e.s.d.'s is used for estimating e.s.d.'s involving l.s. planes.
Refinement. Refinement of *F*^2^ against ALL reflections. The weighted *R*-factor *wR* and goodness of fit *S* are based on *F*^2^, conventional *R*-factors *R* are based on *F*, with *F* set to zero for negative *F*^2^. The threshold expression of *F*^2^ > σ(*F*^2^) is used only for calculating *R*-factors(gt) *etc*. and is not relevant to the choice of reflections for refinement. *R*-factors based on *F*^2^ are statistically about twice as large as those based on *F*, and *R*- factors based on ALL data will be even larger.

### Fractional atomic coordinates and isotropic or equivalent isotropic displacement parameters (Å^2^)

**Table:**

	*x*	*y*	*z*	*U*_iso_*/*U*_eq_	
N1	0.2255 (4)	0.7148 (5)	0.2340 (2)	0.0463 (7)	
H1	0.2726	0.8381	0.2218	0.056*	
N2	0.2198 (3)	0.6193 (5)	0.31994 (19)	0.0469 (8)	
H2	0.2635	0.6724	0.3713	0.056*	
C6	0.3512 (4)	0.7229 (6)	0.5523 (2)	0.0405 (8)	
N3	0.2184 (3)	1.3159 (5)	0.7707 (2)	0.0487 (7)	
N4	0.5550 (4)	0.7118 (5)	0.8914 (2)	0.0484 (8)	
O1	0.5414 (3)	0.5277 (4)	0.70183 (15)	0.0454 (6)	
O2	0.4254 (3)	0.5367 (4)	0.53738 (15)	0.0581 (7)	
H2A	0.4770	0.4990	0.5855	0.087*	
O3	0.2657 (3)	0.8102 (4)	0.48999 (14)	0.0493 (6)	
O4	0.1417 (3)	1.4008 (4)	0.70451 (18)	0.0632 (7)	
O5	0.2271 (3)	1.3968 (5)	0.85007 (18)	0.0689 (8)	
O6	0.5255 (4)	0.7837 (5)	0.96945 (17)	0.0833 (10)	
O7	0.6547 (4)	0.5660 (5)	0.87978 (17)	0.0698 (8)	
C1	0.1300 (5)	0.6449 (8)	0.0686 (2)	0.0717 (13)	
H1A	0.1787	0.7868	0.0584	0.108*	
H1B	0.0155	0.6492	0.0495	0.108*	
H1C	0.1854	0.5360	0.0320	0.108*	
C2	0.1462 (4)	0.5868 (6)	0.1713 (2)	0.0464 (9)	
C3	0.0892 (4)	0.4066 (7)	0.2187 (3)	0.0544 (10)	
H3	0.0298	0.2892	0.1925	0.065*	
C4	0.1370 (4)	0.4321 (6)	0.3134 (2)	0.0464 (9)	
C5	0.1105 (4)	0.2906 (7)	0.3972 (3)	0.0625 (11)	
H5A	0.2148	0.2570	0.4278	0.094*	
H5B	0.0572	0.1562	0.3774	0.094*	
H5C	0.0422	0.3672	0.4406	0.094*	
C12	0.4688 (4)	0.7102 (5)	0.7195 (2)	0.0353 (8)	
C7	0.3732 (3)	0.8231 (6)	0.64774 (19)	0.0339 (7)	
C8	0.2969 (4)	1.0188 (6)	0.6644 (2)	0.0376 (8)	
H8	0.2396	1.0905	0.6154	0.045*	
C9	0.3041 (4)	1.1108 (6)	0.7533 (2)	0.0368 (8)	
C10	0.3900 (4)	1.0088 (6)	0.8269 (2)	0.0396 (8)	
H10	0.3935	1.0711	0.8870	0.048*	
C11	0.4701 (4)	0.8142 (6)	0.8101 (2)	0.0366 (7)	

### Atomic displacement parameters (Å^2^)

**Table:**

	*U*^11^	*U*^22^	*U*^33^	*U*^12^	*U*^13^	*U*^23^
N1	0.0503 (17)	0.0446 (18)	0.0438 (17)	−0.0035 (14)	−0.0020 (13)	−0.0021 (14)
N2	0.0493 (18)	0.051 (2)	0.0397 (16)	−0.0042 (16)	−0.0060 (13)	−0.0055 (16)
C6	0.0382 (18)	0.048 (2)	0.0356 (19)	−0.0017 (17)	0.0045 (16)	−0.0016 (17)
N3	0.0479 (17)	0.0412 (19)	0.057 (2)	0.0007 (16)	0.0029 (15)	−0.0032 (18)
N4	0.064 (2)	0.044 (2)	0.0372 (18)	−0.0012 (16)	−0.0046 (15)	0.0033 (15)
O1	0.0544 (14)	0.0412 (14)	0.0404 (13)	0.0081 (12)	−0.0011 (10)	−0.0056 (11)
O2	0.0701 (17)	0.0628 (18)	0.0408 (14)	0.0203 (15)	−0.0077 (11)	−0.0155 (13)
O3	0.0554 (13)	0.0590 (16)	0.0329 (12)	0.0050 (13)	−0.0072 (11)	0.0004 (12)
O4	0.0679 (17)	0.0510 (17)	0.0699 (17)	0.0183 (14)	−0.0112 (14)	0.0030 (15)
O5	0.088 (2)	0.0582 (18)	0.0602 (17)	0.0083 (16)	0.0045 (14)	−0.0181 (15)
O6	0.144 (3)	0.073 (2)	0.0317 (15)	0.033 (2)	−0.0088 (15)	−0.0034 (14)
O7	0.088 (2)	0.070 (2)	0.0509 (15)	0.0294 (18)	−0.0100 (14)	0.0072 (15)
C1	0.079 (3)	0.093 (3)	0.043 (2)	−0.017 (3)	−0.005 (2)	−0.009 (2)
C2	0.044 (2)	0.052 (3)	0.0424 (19)	0.0036 (19)	−0.0059 (16)	−0.0103 (19)
C3	0.050 (2)	0.056 (3)	0.057 (2)	−0.006 (2)	−0.0065 (18)	−0.023 (2)
C4	0.0363 (19)	0.042 (2)	0.061 (2)	0.0018 (18)	0.0040 (16)	−0.0034 (19)
C5	0.061 (2)	0.057 (3)	0.069 (3)	−0.001 (2)	−0.0008 (19)	0.010 (2)
C12	0.0346 (18)	0.038 (2)	0.0338 (18)	−0.0056 (16)	0.0023 (14)	0.0013 (16)
C7	0.0348 (16)	0.0367 (19)	0.0302 (16)	−0.0059 (16)	0.0012 (13)	0.0009 (15)
C8	0.0393 (19)	0.039 (2)	0.0346 (18)	−0.0019 (17)	−0.0034 (14)	0.0036 (16)
C9	0.0394 (19)	0.0310 (19)	0.0399 (18)	−0.0045 (16)	0.0016 (14)	−0.0010 (16)
C10	0.049 (2)	0.040 (2)	0.0299 (17)	−0.0064 (18)	0.0020 (15)	−0.0014 (16)
C11	0.0403 (18)	0.0381 (19)	0.0312 (17)	−0.0023 (18)	−0.0024 (13)	0.0046 (16)

### Geometric parameters (Å, º)

**Table:**

N1—C2	1.329 (4)	C1—H1B	0.9600
N1—N2	1.348 (4)	C1—H1C	0.9600
N1—H1	0.8600	C2—C3	1.371 (5)
N2—C4	1.321 (4)	C3—C4	1.391 (5)
N2—H2	0.8600	C3—H3	0.9300
C6—O3	1.224 (4)	C4—C5	1.484 (5)
C6—O2	1.301 (4)	C5—H5A	0.9600
C6—C7	1.486 (4)	C5—H5B	0.9600
N3—O4	1.221 (3)	C5—H5C	0.9600
N3—O5	1.226 (3)	C12—C11	1.427 (4)
N3—C9	1.449 (4)	C12—C7	1.431 (4)
N4—O7	1.213 (4)	C7—C8	1.363 (4)
N4—O6	1.218 (3)	C8—C9	1.375 (4)
N4—C11	1.460 (4)	C8—H8	0.9300
O1—C12	1.283 (4)	C9—C10	1.379 (4)
O2—H2A	0.8200	C10—C11	1.372 (4)
C1—C2	1.496 (5)	C10—H10	0.9300
C1—H1A	0.9600		
			
C2—N1—N2	108.7 (3)	N2—C4—C3	106.7 (3)
C2—N1—H1	125.6	N2—C4—C5	121.9 (3)
N2—N1—H1	125.6	C3—C4—C5	131.4 (3)
C4—N2—N1	109.8 (3)	C4—C5—H5A	109.5
C4—N2—H2	125.1	C4—C5—H5B	109.5
N1—N2—H2	125.1	H5A—C5—H5B	109.5
O3—C6—O2	120.9 (3)	C4—C5—H5C	109.5
O3—C6—C7	121.7 (3)	H5A—C5—H5C	109.5
O2—C6—C7	117.4 (3)	H5B—C5—H5C	109.5
O4—N3—O5	123.1 (3)	O1—C12—C11	124.5 (3)
O4—N3—C9	117.9 (3)	O1—C12—C7	121.0 (3)
O5—N3—C9	119.0 (3)	C11—C12—C7	114.4 (3)
O7—N4—O6	122.4 (3)	C8—C7—C12	122.2 (3)
O7—N4—C11	120.2 (3)	C8—C7—C6	118.1 (3)
O6—N4—C11	117.4 (3)	C12—C7—C6	119.7 (3)
C6—O2—H2A	109.5	C7—C8—C9	120.4 (3)
C2—C1—H1A	109.5	C7—C8—H8	119.8
C2—C1—H1B	109.5	C9—C8—H8	119.8
H1A—C1—H1B	109.5	C8—C9—C10	120.8 (3)
C2—C1—H1C	109.5	C8—C9—N3	119.8 (3)
H1A—C1—H1C	109.5	C10—C9—N3	119.4 (3)
H1B—C1—H1C	109.5	C11—C10—C9	119.1 (3)
N1—C2—C3	107.6 (3)	C11—C10—H10	120.5
N1—C2—C1	122.4 (4)	C9—C10—H10	120.5
C3—C2—C1	130.0 (3)	C10—C11—C12	123.1 (3)
C2—C3—C4	107.2 (3)	C10—C11—N4	116.3 (3)
C2—C3—H3	126.4	C12—C11—N4	120.6 (3)
C4—C3—H3	126.4		
			
C2—N1—N2—C4	−0.4 (4)	C7—C8—C9—C10	0.8 (5)
N2—N1—C2—C3	0.0 (4)	C7—C8—C9—N3	−178.3 (3)
N2—N1—C2—C1	−179.8 (3)	O4—N3—C9—C8	0.0 (4)
N1—C2—C3—C4	0.3 (4)	O5—N3—C9—C8	−179.4 (3)
C1—C2—C3—C4	−179.9 (4)	O4—N3—C9—C10	−179.1 (3)
N1—N2—C4—C3	0.6 (3)	O5—N3—C9—C10	1.5 (4)
N1—N2—C4—C5	−179.9 (3)	C8—C9—C10—C11	0.6 (5)
C2—C3—C4—N2	−0.6 (4)	N3—C9—C10—C11	179.7 (3)
C2—C3—C4—C5	180.0 (4)	C9—C10—C11—C12	−0.2 (4)
O1—C12—C7—C8	−179.0 (3)	C9—C10—C11—N4	−178.4 (3)
C11—C12—C7—C8	2.9 (4)	O1—C12—C11—C10	−179.5 (3)
O1—C12—C7—C6	2.8 (4)	C7—C12—C11—C10	−1.4 (4)
C11—C12—C7—C6	−175.3 (3)	O1—C12—C11—N4	−1.3 (5)
O3—C6—C7—C8	−0.9 (4)	C7—C12—C11—N4	176.7 (3)
O2—C6—C7—C8	179.7 (3)	O7—N4—C11—C10	−165.3 (3)
O3—C6—C7—C12	177.4 (3)	O6—N4—C11—C10	12.4 (4)
O2—C6—C7—C12	−2.1 (4)	O7—N4—C11—C12	16.5 (5)
C12—C7—C8—C9	−2.7 (5)	O6—N4—C11—C12	−165.9 (3)
C6—C7—C8—C9	175.5 (3)		

### Hydrogen-bond geometry (Å, º)

**Table:**

*D*—H···*A*	*D*—H	H···*A*	*D*···*A*	*D*—H···*A*
N1—H1···O7^i^	0.86	2.09	2.859 (4)	148
N1—H1···O1^i^	0.86	2.16	2.809 (4)	132
N2—H2···O3	0.86	1.88	2.684 (3)	156
O2—H2*A*···O1	0.82	1.72	2.481 (3)	154
C1—H1*A*···O7^i^	0.96	2.32	3.166 (5)	147
C5—H5*B*···O4^ii^	0.96	2.49	3.414 (5)	160
C10—H10···O6^iii^	0.93	2.48	3.379 (4)	164

Symmetry codes: (i) −*x*+1, *y*+1/2, −*z*+1; (ii) −*x*, *y*−3/2, −*z*+1; (iii) −*x*+1, *y*+1/2, −*z*+2.

## References

[bb1] Bruker (2002). *SADABS*, *SMART* and *SAINT* Bruker AXS Inc., Madison, Wisconsin, USA.

[bb2] Jin, S. W., Zhang, W. B., Liu, L., Gao, H. F., Wang, D. Q., Chen, R. P. & Xu, X. L. (2010). *J. Mol. Struct.* **975**, 128–136.

[bb3] Sheldrick, G. M. (2008). *Acta Cryst.* A**64**, 112–122. 10.1107/S0108767307043930 18156677

[bb4] Smith, G., Wermuth, U. D., Healy, P. C. & White, J. M. (2011). *J. Chem. Crystallogr.* **41**, 1649–1662.

